# Supplementary material for: A Likelihood Approach for Real-Time Calibration of Stochastic Compartmental Epidemic Models
Source: PLoS Comput Biol. 2017 Jan 17;13(1):e1005257. doi: 10.1371/journal.pcbi.1005257 (PMC5240920; doi:10.1371/journal.pcbi.1005257)
Supplement: S1 File — (TAR.GZ) [file pcbi.1005257.s014.tar.gz › HSPH_Online-SI-Revision/IPoi09/n1-extreme/n1-extreme_BENCH_summary.pdf]

peak -8	R0	$1/\theta_2$	inf	inflog	newly	newlylog	Reff	AttackRate
mean	26.1	31.3	127.2	0.6	232.	0.7	26.9	37.
median	24.5	27.7	86.8	0.6	95.8	0.6	26.	35.5
Quantile .1	16.2	17.2	61.3	0.4	66.4	0.4	16.	13.2
Quantile .9	38.7	46.6	197.4	1.	320.5	1.	41.8	62.8
28	38	26						
length	50							

peak -4	R0	$1/\theta_2$	inf	inflog	newly	newlylog	Reff	AttackRate
mean	18.3	29.3	72.3	0.3	62.6	0.3	27.7	11.1
median	14.6	20.7	56.2	0.3	54.	0.3	26.9	8.6
Quantile .1	8.6	10.9	31.1	0.2	25.5	0.1	15.4	4.3
Quantile .9	31.6	59.6	142.3	0.5	117.2	0.4	41.5	18.5
25	28	31						
length	50							

peak	R0	$1/\theta_2$	inf	inflog	newly	newlylog	Reff	AttackRate
mean	22.8	27.9	42.2	0.2	32.2	0.2	46.6	11.4
median	21.1	18.4	29.	0.1	27.7	0.1	42.5	7.2
Quantile .1	2.7	2.2	17.1	0.1	19.3	0.1	32.8	1.3
Quantile .9	44.9	69.	83.6	0.3	44.4	0.2	54.4	20.9
2	4	11						
length	50							

peak +4	R0	$1/\theta_2$	inf	inflog	newly	newlylog	Reff	AttackRate
mean	24.5	29.5	68.5	0.3	48.8	0.3	46.3	11.8
median	21.	19.9	48.8	0.2	44.5	0.2	40.2	7.1
Quantile .1	5.3	2.1	33.6	0.1	31.1	0.1	28.7	1.4
Quantile .9	52.9	72.7	138.3	0.4	66.9	0.3	59.4	22.9
0	4	11						
length	50							

peak +8	R0	$1/\theta_2$	inf	inflog	newly	newlylog	Reff	AttackRate
mean	24.3	29.3	115.5	0.3	91.4	0.3	40.	11.8
median	22.3	19.4	73.2	0.3	69.4	0.3	34.9	7.
Quantile .1	3.2	1.9	46.7	0.2	47.5	0.2	20.1	1.3
Quantile .9	53.	73.9	249.	0.5	161.9	0.4	56.9	22.7
0	1	9						
length	50							
